# Physiological Responses of European Seabass (*Dicentrarchus labrax*) Juveniles to Long-Term Feeding with Increasing Levels of EPA and DHA: Effects of Thermal Challenge

**DOI:** 10.3390/ani16193119

**Published:** 2026-10-04

**Authors:** Lúcia Vieira, Diogo Amaral, Bruno Silveira, Thaís Cavalheri, Filipa Fontinha, Narcisa Bandarra, Sara Magalhães, Tiago Aires, Rodrigo O. A. Ozório, Aires Oliva-Teles, Helena Peres, Rui Magalhães

**Affiliations:** 1 CIIMAR/CIMAR LA, Interdisciplinary Center of Marine and Environmental Research, University of Porto, Terminal de Cruzeiros do Porto de Leixões, 4450-208 Matosinhos, Portugal; damaral@ciimar.up.pt (D.A.); thais.oliveira@ciimar.up.pt (T.C.); ffontinha@ciimar.up.pt (F.F.); rodrigo.ozorio@ciimar.up.pt (R.O.A.O.); aoteles@fc.up.pt (A.O.-T.); pereshelena@fc.up.pt (H.P.); rmagalhaes@ciimar.up.pt (R.M.); 2 Department of Biology, Faculty of Science, University of Porto, Rua do Campo Alegre, 4169-007 Porto, Portugal; 3 School of Medicine and Biomedical Sciences, University of Porto, Rua de Jorge Viterbo Ferreira 228, 4050-313 Porto, Portugal; 4 IPMA—Portuguese Institute for Sea and Atmosphere, 1495-006 Lisboa, Portugal; 5 Sorgal S.A., Sociedade de Óleos e Rações, EN109 Pardala, 3880-728 São João Ovar, Portugal

**Keywords:** long-chain polyunsaturated fatty acids, marine aquaculture, growth performance, welfare, climate change

## Abstract

Fish oil has traditionally been the primary source of n-3 long-chain polyunsaturated FA (n-3 LC-PUFA) in aquafeeds but is increasingly being replaced by alternative lipid sources. However, marine carnivorous fish require dietary n-3 LC-PUFA, particularly eicosapentaenoic acid (EPA) and docosahexaenoic acid (DHA), which these alternative sources lack. This study assessed the long-term impact of graded levels of dietary EPA+DHA from microalgal oil on European seabass growth, feed utilization, immunological and oxidative status, and muscle FA profile. Six isoproteic (47% protein) and isolipidic (17% lipid) diets containing 0.56%, 0.66%, 0.71%, 0.90%, 1.11%, and 1.27% EPA+DHA were fed to fish (107 g initial weight) for 180 days at 23 °C, followed by a 7-day thermal challenge at 30 °C. At the end of the feeding trial, growth performance, feed utilization, organosomatic indices, blood and innate immune parameters, and oxidative stress were unaffected. Muscle FA composition mirrored that of the diets, and linear correlations were observed between muscle and dietary n-3 LC-PUFA levels. In the thermal challenge, no mortalities occurred, but hematological parameters, immune responses, and oxidative stress were intensified. Muscle FA composition remained unchanged, indicating that a dietary EPA+DHA level of 0.56% appears sufficient to meet physiological needs during an acute temperature challenge.

## Simple Summary

Fish oil is an important source of omega-3 fatty acids (FAs) in aquaculture but is increasingly being replaced by alternative oils. Since carnivorous fish need EPA and DHA for normal development and health, identifying suitable dietary levels is essential. In this study, European seabass were fed diets containing increasing levels of EPA+DHA for 180 days. Fish performance, feed efficiency, immune function, and oxidative status were not affected by the dietary treatments. The FA composition of fillets reflected that of the diets. Fish were then exposed to a 7-day thermal challenge at 30 °C. Although this challenge increased immune and oxidative responses, no mortality occurred, and fillet EPA+DHA levels were maintained, indicating that exposure to 30 °C did not compromise fillet EPA and DHA content.

## 1. Introduction

The growing global human population is increasing the demand for seafood. While aquaculture is a viable option to ensure high-quality seafood for human diets, a notable constraint is the limited supply of feed ingredients for aquafeed manufacturing due to competition with direct human consumption and other industries [1]. This raises significant economic concerns, since fish oil (FO) prices have increased over the last decade [2]. This has led to partial replacement of FO in aquafeeds by alternatives such as plant oils and oils from processed animal products [3]. This FO replacement, however, is achieved at the expense of reduced dietary n-3 LC-PUFA content, as alternative oils are notably low in EPA and DHA [4].

Microalgal oils are a promising alternative to FO, as microalgae are notable producers of essential fatty acids (EFAs) [5]. However, EPA and DHA ratios in microalgae oils can differ substantially from those in FO, potentially affecting their physiological utilization by fish. For instance, while FO has a roughly balanced ratio of EPA and DHA, with EPA prevailing in most cases, microalgae oils have much lower EPA levels, which are often vestigial [6]. Many algal oils have already been used as alternatives to FO, such as *Schizochytrium* sp. and *Ulkenia* spp., with EPA levels ranging from 2% to 5% and DHA levels ranging from 18% to 30% [7,8]. In this context, Veramaris oil stands out for its higher EPA content (10%). However, the DHA content is still much higher (40%), and the DHA/EPA ratio is higher than in most FO sources [2].

For most marine and high-trophic-level fish species, n-3 LC-PUFAs are regarded as EFAs because the fish are unable to synthesize them efficiently from precursors (α-linolenic acid, ALA). These LC-PUFAs are key constituents of cellular membrane phospholipids and play critical roles in several physiological processes, including membrane fluidity and function, cellular signaling, and inflammatory responses [9]. Additionally, LC-PUFAs play a significant role in vision and nervous system function [10]. Given their pivotal involvement in ontogenic processes, disease resistance, reproductive success, lipid metabolism, immune function, and resilience to stress, maintaining sufficient tissue concentrations of n-3 LC-PUFAs is imperative for growth performance and for preserving fish health and welfare [11,12]. Moreover, an adequate balance of EFA may be relevant for zootechnical performance and for optimizing immune status [13,14].

Beyond fish physiological requirements, fillet EPA and DHA content is a determinant factor of the harvested end-product [15], since consumers value food rich in EPA and DHA due to their role in promoting cardiovascular health, supporting brain and visual development, and exerting anti-inflammatory, anticarcinogenic, and neuroprotective effects [16].

Climate change has a direct impact on all aspects of society and industry, including aquaculture production. One such impact is global warming, which has led to a steady increase in average ocean temperatures. In the case of the Mediterranean Sea, the average water temperature has been rising over the years, with the highest daily median temperature reaching 28.9 °C in 2024, during a marine heatwave [17], and the highest recorded surface temperature reaching 31.9 °C on the Spanish Mediterranean coast, also in 2024 [18]. Furthermore, an intensification in frequency, intensity, and duration of marine heatwaves has been observed in recent years [19]. As ectotherms, fish physiology is heavily influenced by water temperature, as it affects their metabolic rate, nutrient digestibility and utilization, growth performance, whole-body composition, fillet FA profile, and shelf-life [20,21,22,23,24]. Additionally, fish welfare and health status may decline when environmental temperature rises outside the species’ optimal range, as unfavorable conditions undermine homeostasis and trigger stress responses, including biochemical, physiological, immunological, and behavioral responses [25]. Thus, stressful production conditions will, in the long term, result in reduced fish growth and compromised disease resistance of aquaculture fish stocks [26].

European seabass (*Dicentrarchus labrax*) is primarily produced in the Mediterranean region, which accounts for nearly 94% of European seabass aquaculture production [27], and holds a prominent position in European aquaculture, ranking third in production behind Atlantic salmon (*Salmo salar*) and rainbow trout (*Oncorhynchus mykiss*) [28].

The projected increase in Mediterranean seawater temperatures during the summer months will expose European seabass to conditions exceeding its upper thermal tolerance threshold of approximately 28 °C [29], making it increasingly susceptible to lower growth performance, increased stress and disease vulnerability, and greater economic risk.

The objective of this study was to determine the impact of dietary EFA levels on the performance and resilience of marine fish exposed to periods of high water temperature. Accordingly, the present study evaluates the long-term effects of dietary EPA+DHA levels on growth performance, feed utilization, survival, muscle FA composition, immune parameters, and oxidative status in European seabass juveniles reared under optimal conditions and subsequently exposed to an acute high-temperature challenge.

## 2. Materials and Methods

### 2.1. Ethics Statement

All experimental procedures were performed in accordance with FELASA category C recommendations and European Union Directive 2010/63/EU on the protection of animals used for scientific purposes and were approved by the Ethics Committee of CIIMAR (ORBEA_CIIMAR_27_2019).

All biological waste generated during the experiment was discarded as Group IV healthcare waste and was handled in accordance with the applicable CIIMAR procedures.

### 2.2. Experimental Diets

Six experimental diets were formulated to be isoproteic (47% crude protein) and isolipidic (17% crude lipid) and to include 2.5% salmon by-product oil and increasing levels of Veramaris^®^ microalgal oil (0% to 1.75%) replacing rapeseed oil, to provide from 0.56% to 1.27% of EPA+DHA in the diet.

All dietary ingredients were finely ground, mixed, and dry-pelleted through a 3 mm matrix in a laboratory pellet mill (California Pellet Mill, Crawfordsville, IN, USA). Pellets were dried at 40 °C for 48 h and stored at −20 °C until further use. Diet composition and proximate analyses are presented in Table 1.

### 2.3. Feeding Trial

European seabass juveniles were transported from a commercial fish farm (Atlantik Fish, Castro Marim, Portugal) to CIIMAR, Matosinhos, Portugal. Fish were first kept in quarantine for 30 days and then moved to an experimental recirculating water system with 18 fiberglass tanks of 500 L capacity for acclimatization to the rearing conditions for 30 days before the start of the trial. The system was thermoregulated at 23 ± 1 °C, and a continuous flow of filtered seawater was maintained (water flow: 2.5–3.5 L min^−1^; salinity: 33 ± 1‰; dissolved oxygen: 7 ± 1 mg L^−1^; pH: 7 ± 1; nitrogenous compounds: <0.05 mg L^−1^). The photoperiod was set to 12 h light: 12 h dark (8:00–20:00). At the beginning of the trial, 12 fish (initial body weight: 107 g) were randomly distributed to each tank, and the experimental diets were also randomly assigned to triplicate tanks. During the trial, fish were hand-fed twice per day until apparent visual satiation, 6 days a week, over 180 days, from November to May.

### 2.4. Temperature Challenge

After the growth trial ended, the water temperature was raised for 2 days until reaching 30 °C and maintained for 7 days. During this period, the fish were fed to satiety with the same diets; dissolved oxygen was kept at 7 ± 1 mg L^−1^, salinity at 33 ± 1‰, and nitrogenous compounds below 0.05 mg L^−1^. Mortality and signs of distress or disease were checked every day.

### 2.5. Sampling

At the end of the feeding trial and the temperature challenge, fish in each tank were lightly anesthetized with 0.3 mL L^−1^ of 2-phenoxyethanol and bulk-weighed after one day of feed deprivation. Fish were then fed for two more days to allow recovery from the final weighing manipulation stress. At the end of the growth trial and the temperature challenge, 3 fish from each tank were randomly selected, and 2 mL of blood was collected from the caudal vein using heparinized syringes, 4 h after the morning meal, according to FAO (1995) [30]. Blood was then centrifuged at 10,000× *g* for 10 min, and plasma aliquots were frozen at −80 °C until analyzed. Fish were then euthanized by decapitation, following the Directive 2010/63/EU of the European Parliament and of the Council of 22 September 2010 on the protection of animals used for scientific purposes, explicitly following the guidelines outlined in Chapter I, Article 6, and Annex IV. Wet weight, liver weight, and viscera weight were also quantified for hepatosomatic and visceral index determination. Then, the liver of three fish per tank was collected for analysis of oxidative stress indicators, and the fillet was collected for FA profile quantification; these were stored at −80 °C until analyzed.

### 2.6. Blood Parameters

Blood parameters were determined according to Magalhães et al. [13]. Fresh heparinized blood was used to determine hematocrit (HT) by microcentrifugation of hematocrit tubes at 10,000× *g* for 10 min at room temperature. Hemoglobin (HB) was determined using Drabkin’s solution (SPINREACT kit, ref. 1001230, Spinreact, Girona, Spain), following the manufacturer’s instructions. Briefly, 4 µL of sample was mixed with 1 mL of the Drabkin solution provided by the kit, and the absorbance was measured at 540 nm.

Plasma metabolites concentration was determined spectrophotometrically using commercial kits for lactate (D-/L-Lactic acid, AK00141, NZYTech Ltd., Lisbon, Portugal) and glucose (TR, GOD-POD, 61269/61270, Spinreact, Girona, Spain). Briefly, for lactate determination, 10 µL of sample was mixed with 1.5 mL of distilled water and the solutions provided by the kit, according to the manufacturer’s instructions. The absorbance was measured at 340 nm. For glucose determination, 10 µL of sample was incubated with 1 mL of solution provided by the kit for 10 min at 37 °C, and absorbance was measured at 505 nm.

### 2.7. Plasma Innate Immune Parameters

Lysozyme was measured by a turbidimetric assay based on the lysis of *Micrococcus lysodeikticus*, using chicken egg white lysozyme as a standard [31]. Peroxidase activity was determined by 3,3′,5,5′-tetramethylbenzidine hydrochloride reduction as described by Quade et al. [32]. Hemolytic activity of the alternative complement pathway (ACH50) was assessed through defibrinated sheep red blood cells (E&O Laboratories Limited, North Ayrshire, Scotland) agglutination, as described by Sunyer et al. [33], and determined based on the concentration of plasma inducing the hemolysis of 50% of sheep blood cells (ACH_50_ mL^−1^).

### 2.8. Chemical Analysis

Ingredients and diets’ chemical analysis were determined according to AOAC [34] methods (Table 1) as follows: dry matter by drying samples in an oven at 100 °C for 24 h, ash by combustion in a muffle furnace at 450 °C for 16 h, crude protein (N × 6.25) by the Kjeldahl method using Kjeltec digestion and Velp distillation units (Tecator Systems, Höganäs, Sweden; model 1015 and Velp, Usmate, Italy, model UDK 149 Semi-automatic, respectively), crude lipids by petroleum ether extraction in a Soxtec system (Tecator Systems, Höganäs, Sweden; extraction unit model ST 243 and control unit model CU 2046), and gross energy by direct combustion of diet samples in an adiabatic bomb calorimeter (PARR Instruments, Moline, IL, USA; PARR model 1261).

Dietary and fillet FA profiles were determined according to Bandarra et al. [35]. Briefly, 0.3 g of freeze-dried samples were mixed with a 5 mL of acetyl chloride–methanol solution (1:19, *v*/*v*) and then esterified for 1 h at 80 °C. Then, a mixture of n-heptane:water (2:1 *v*/*v*) was added and centrifuged at 2150× *g* for 10 min. Then, once the organic phase was collected, solvent was removed under nitrogen and the methyl esters were solubilized in 0.1 mL of n-heptane. FA methyl esters (FAMEs) quantification was then carried out by gas chromatography using a CP 3800 Varian Star gas chromatograph (Walnut Creek, CA, USA) (Table 2).

### 2.9. Determination of Hepatic Enzymatic and Non-Enzymatic Oxidative Stress Indicators

Liver samples were homogenized (1:6, *w*/*v*) in ice-cold K-phosphate buffer solution (pH 7.4, 0.1 M). Samples were centrifuged at 30,000× *g* for 30 min at 4 °C and stored at −80 °C.

Soluble protein concentration was assessed according to Bradford [36] using the Sigma-Aldrich protein assay kit and bovine serum albumin as a standard. Catalase (CAT; EC 1.11.1.6) activity was determined by measuring the decrease in hydrogen peroxide concentration, according to Aebi [37]. Glutathione reductase (GR; EC 1.6.4.2) activity was analyzed by measuring NADPH oxidation as reported by Morales et al. [38]. Glutathione peroxidase (GPX; EC 1.11.1.9) activity was determined as described by Flohé et al. [39], by measuring the NADPH consumption rate resulting from the oxidation of reduced glutathione (GSH) to oxidized glutathione (GSSG). Superoxide dismutase (SOD; EC 1.15.1.1) activity was assayed by the ferricytochrome method, as described by McCord et al. [40], using xanthine/xanthine oxidase as the source of superoxide radicals. All enzymatic assays were performed at 37 °C in a Multiskan GO microplate reader (Model 5111 9200; Thermo Scientific, Nanjing, China).

Lipid peroxidation (LPO) was assessed through malondialdehyde (MDA) concentration as described by Buege et al. [41]. Briefly, in the presence of thiobarbituric acid, MDA reacts, producing colored thiobarbituric acid-reactive substances (TBARSs). MDA was used as a standard, and values were measured at 535 nm.

### 2.10. Statistical Analysis

Statistical analysis was performed using SPSS 29.0.0 for Windows (IBM, SPSS, Statistics, New York, NY, USA) following Dawson and Trapp (2004) [42]. Data were checked for normality and homogeneity of variances and normalized when required. Statistical analysis of the zootechnical performance and fatty acid profile data was performed using one-way analysis of variance (ANOVA) and polynomial contrasts, while hematological, plasma, hepatic oxidative stress, enzymatic parameters, and the sum of SFA, MUFA, and PUFA were analyzed using two-way analysis of variance with diet and temperature as independent variables, followed by polynomial contrasts. The Tukey multiple range test was applied to detect significant differences among means (*p* < 0.05).

## 3. Results

European seabass juveniles accepted all diets well, and no signs of disease were observed during the growth trial. Survival was high and not affected by diet composition (Table 3). Growth performance, feed efficiency, and protein efficiency ratio were unaffected by diet composition. Hepatosomatic and visceral indices were also unaffected by dietary composition. No mortality was observed during the 7-day temperature challenge.

Hematocrit showed a quadratic response to dietary EPA+DHA content, with the lowest values observed in fish fed the 0.9% and 1.1% EPA+DHA diets (*p*-value = 0.008) (Table 4). MCHC showed an inverse response, with the highest values being observed in the same diets (*p*-value = 0.002). The acute temperature challenge increased hemoglobin (*p*-value = 0.001), MCHC (*p*-value = 0.001), and glucose content (*p*-value = 0.001) and peroxidase (*p*-value = 0.001) and ACH50 activities (*p*-value = 0.007), while hematocrit decreased (*p*-value = 0.014). Lactate and lysozyme were not affected by dietary treatments or temperature.

Hepatic LPO, CAT, and SOD activities were unaffected by diet composition. However, LPO was higher in fish submitted to the acute thermic challenge (*p*-value = 0.026) (Table 5). GR activity was also higher following the thermic challenge and showed a quadratic response to diet composition, with the highest values observed in fish fed the 0.9% EPA+DHA diet (*p*-value = 0.001). GPX activity was also higher in the thermally challenged fish and was affected by diet composition only in the thermally challenged fish (interaction *p*-value = 0.006).

At the end of the feeding trial, muscle FA profile was modulated by the dietary treatments (Table 6). Muscle LA and ALA decreased while ARA and the n3/n6 ratio increased with the increasing EPA+DHA levels (*p*-value = 0.001). SFA were highest in fish fed the 0.71 EPA+DHA diet (*p*-value = 0.001), while MUFA decreased linearly with dietary EPA+DHA levels (*p*-value = 0.001). On the other hand, PUFA, EPA, and DHA increased linearly with dietary EPA+DHA levels (*p*-value = 0.001). The acute temperature challenge did not affect the muscle FA composition (Table 6).

The atherogenicity (*p*-value = 0.001) and thrombogenicity indices (*p*-value = 0.012) showed the highest values, and the hypo-hypercholesterolemic index (*p*-value = 0.001) showed the lowest value in fish fed the 0.71% EPA+DHA diet; no further differences were observed among the other groups (Table 6). The peroxidation index and the nutritional contribution of the fillets to the daily intake of EPA and DHA (NC_250_) increased linearly with dietary EPA and DHA levels (*p*-value = 0.001).

**Figure 1:**
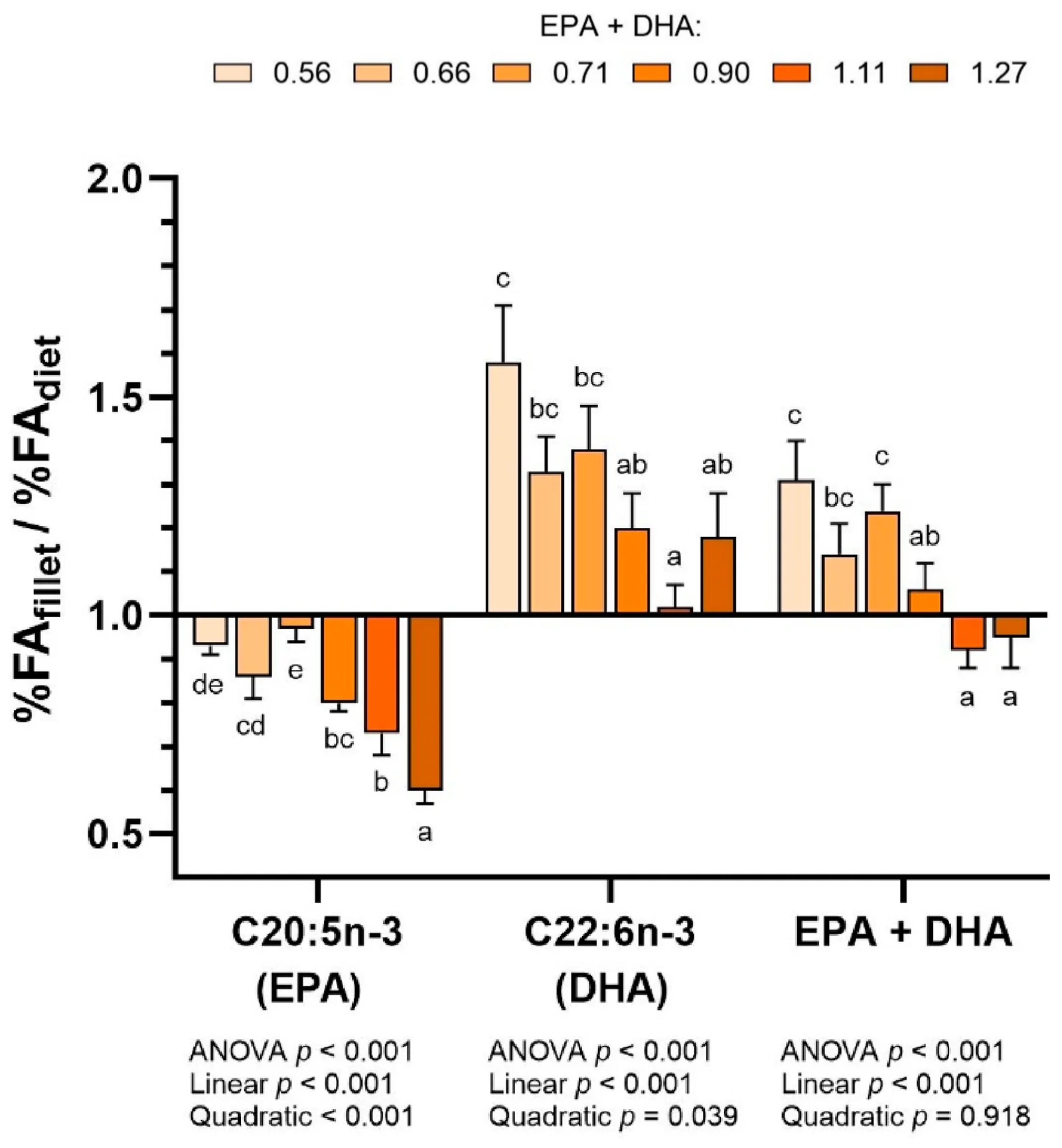
Fillet-to-diet fatty acid ratios for European seabass fed the experimental diets for 180 days. Values are means of triplicate groups; different letters indicate significant differences (*p* < 0.05).

The fillet/diet ratios of EPA and DHA are represented in Figure 1. The fillet/diet EPA ratio was lower than 1 and decreased linearly with dietary EPA+DHA content, whereas the opposite was observed for the DHA ratio.

## 4. Discussion

In this study, no differences in growth performance were observed among fish fed graded dietary EPA+DHA, indicating that dietary requirements for European seabass weighing from 100 g to 460 g were fulfilled with 0.56% EPA+DHA in the diet (corresponding to 4.7% dietary FA). Previously, a dietary EPA+DHA requirement of 0.7% was indicated for juveniles weighing 14–26 g (corresponding to 3.8% dietary FA) [44]. Recently, in a study in which fish oil (FO) was replaced by vegetable oils (VOs) at a gradient of EPA+DHA levels, the growth performance of seabass weighing 3–150 g was negatively affected by the diet containing 0.3% EPA+DHA, whereas no negative effects on growth performance were observed with diets containing EPA+DHA above 0.88% (corresponding to 3.85% dietary FA) [45].

In aquafeeds, FO is the traditional source of EPA+DHA, and previous studies have evaluated replacing FO with alternative oil sources in European seabass juveniles. Replacing FO with alternative marine oil sources [46] or vegetable oils [47] in diets containing EPA+DHA above 0.8% was feasible and did not affect seabass growth performance. A blend of land animal fats could also replace up to 75% of FO (corresponding to a diet including 0.97% EPA+DHA) without affecting the growth performance of seabass juveniles [48]. However, growth performance was significantly decreased with a 100% replacement of FO, corresponding to a diet containing 0.38% EPA+DHA, a value well below the requirement established in the present study. However, in a study testing a combined replacement of FM and FO in diets for seabass juveniles, growth performance was reduced in diets including less than 10% FM and 3% FO, although dietary EPA+DHA was above 1%, potentially due to lower feed intake of European seabass [49]. The reduced growth performance observed in seabass fed EPA+DHA at 4.7% FA might be due to the DHA/EPA ratio of 0.7 of the experimental treatment [50], which is in the lower end of the recommended range of DHA/EPA ratio values (0.5–2.0) required for normal growth and development of marine fish species [51]. Furthermore, the previous study was carried out in fingerlings with an initial body weight of 9 g, further suggesting that European seabass exhibit increasing tolerance to lower EPA+DHA levels as they grow, with fish in early life stages being more sensitive than larger fish [52].

The HSI and VSI indices were not affected by the diets, indicating an adequate hepatic function. Similar results were also reported in European seabass fed dietary EPA+DHA levels ranging from 1.60% to 2.60% [46] and in fish fed diets containing 0.4% or 2.2% of n-3 LC-PUFA [53]. In contrast, other studies reported increased HSI with dietary EPA+DHA inclusion levels ranging from 0.4% to 2.8% [47,48,49] and reduced HSI with dietary EPA+DHA inclusion levels from 0.30% to 4.80% [45].

In the present study, plasma glucose, lactate, and HB were unaffected by graded levels of EPA+DHA. Similar results were also observed in gilthead seabream [11], European seabass [47], black seabream (*Acanthopagrus schlegelii*) [54], and rohu (*Labeo rohita*) [55]. Data on the effects of dietary EPA+DHA levels on hematocrit (HT) are less consistent. In this study, HT showed a negative quadratic trend with increasing dietary EPA+DHA levels. In contrast, in black seabream and rainbow trout [56], a linear increase was observed [54], while no effects were observed in gilthead seabream [11] and rohu (*Labeo rohita*) [55].

Increased plasma glucose and lactate levels are considered a physiological stress biomarker [57]. Accordingly, in the present study, plasma glucose levels were higher in fish subjected to the thermal challenge. Similarly, in another study in which European seabass were exposed to temperatures of 20, 23, 26, 29, and 31 °C, plasma glucose and cortisol levels were higher at 31 °C than at the other temperatures [58]. Overall, these results suggest that 29 °C can be considered a threshold temperature above which fish become thermally stressed.

Thermal stress also leads to an increase in red blood cells due to the higher demand for oxygen transport and, therefore, to an increase in HB, HT, and MCHC [59], as observed in Nile tilapia reared from 31 °C to 34 °C [60] and consistent with the results from the present study. Lactate, however, was not elevated by the high-water-temperature challenge. As lactate is produced under stress due to anaerobic metabolism, the lack of lactate increase at higher temperatures may be due to the maintenance of a high dissolved oxygen concentration in the system, which may have helped fish resistance to heat stress [61].

The innate immune parameters measured in this study (peroxidase, lysozyme, and ACH50) were not affected by EPA+DHA levels. Similarly, in another study on European seabass and gilthead seabream, lysozyme and ACH50 activities were not affected by decreasing dietary EPA+DHA [62,63]. In contrast, lysozyme activity increased with dietary DHA+EPA levels in Japanese seabass (*Lateolabrax japonicus*), large yellow croaker (*Larimichthys crocea*) [64], and golden pompano (*Trachinotus ovatus*) [65].

High water temperatures outside the species’ optimal range may directly affect the fish’s immune response [66]. Accordingly, in the present study, peroxidase activity and ACH50 increased at the end of the high-temperature challenge, while lysozyme activity was unaffected. The modulation of the immune system in response to high temperature is not yet clear. While some studies have shown that the immune system of fish is enhanced [67,68,69], others have reported that it can be suppressed [70], and others have reported that it is not affected [71].

In this study, hepatic LPO was not affected by dietary EPA+DHA levels, whereas GR and GPX activities decreased with increasing EPA+DHA levels from 0.9% to 1.27%. In other studies, hepatic biomarkers were not affected by increasing EPA+DHA levels, such as observed in black seabream (*Acanthopagrus schlegelii*) [50], largemouth bass (*Micropterus salmoides*) [72], hybrid grouper (*Epinephelus fuscoguttatus* ♀ × *E. lanceolatus* ♂) [73], and Siberian sturgeon (*Acipenser baerii*) [74]. On the other hand, LPO has been shown to increase with increasing EPA+DHA levels, as observed in gilthead seabream [14], due to PUFA susceptibility to peroxidation [75]. Nonetheless, in the present study, hepatic LPO remained unaffected because DHA/EPA was lower than in the previous study.

Exposure to high water temperatures commonly disrupts fish redox status [76]. Accordingly, in the present study, GR and GPX activities increased at the higher temperature, whereas CAT and SOD activities were not affected by the thermal challenge. Overall, this led to increased LPO levels at the end of the temperature challenge. Although LC-PUFAs are more prone to oxidation than saturated FAs, in the present study, increasing dietary EPA+DHA did not affect hepatic LPO, which remained unaffected by diet composition in fish submitted to the thermal challenge. Likewise, in a previous study, European seabass fed diets containing 0.73% and 1.65% EPA+DHA showed increased hepatic CAT and SOD activities and LPO levels at 20 °C compared with those fed the same diets at 15 °C [77]. However, at both temperatures, SOD activity was higher in fish fed the diet with lower EPA+DHA inclusion (0.73%). Also in European seabass, an increase in antioxidant enzymes and LPO was observed in the liver of fish reared in increasing temperatures from 20 °C to 31 °C for 7 days [57], in the liver of fish reared at 18 °C and 25 °C for 30 days [78], and in the muscle of fish reared at 18 and 24 °C for 30 days [79].

Overall, the muscle FA profiles mirrored those of the diets. These results are consistent with other studies on European seabass [50,80] and with other fish species [11,65]. However, the fillet-to-diet ratios of EPA and DHA decreased as the dietary content of these FAs increased, indicating a metabolic redirection of these more available FAs toward oxidation [10]. Moreover, muscle retention of DHA was higher than that of EPA, reflecting the structural role of DHA as an essential structural component of membrane phospholipids [81]. DPA muscle retention was low and similar to the diets, suggesting that no elongation from EPA or oxidation from DHA occurred [82].

The results of this study also showed that the recommended human daily intake of 250 mg of EPA and DHA [43] can be met by consuming 100 g of fillet, even when produced with the lowest EPA+DHA diet tested (diet 0.56% EPA+DHA). However, fish fed the 1.27% EPA+DHA diets allow the weekly EFSA intake recommendation for EPA+DHA to be satisfied by eating 100 g of fillets only 3 times per week. From a consumer health perspective, beyond n-3 PUFA content, lipid quality indicators, such as AI, TI, and h/H ratio, are also important and were affected by the experimental diets. AI and TI are indicators of the potential impact of food on cardiovascular health. AI is defined by the ratio between the sum of the main SFA and MUFA + PUFA, the former being considered proatherogenic, while TI indicates a propensity for clot formation in blood vessels, and it is defined as the relationship between the prothrombogenetic (SFA) and the antithrombogenetic FA (MUFA + PUFA n-6 + PUFA n-3). Furthermore, the hH ratio compares hypocholesterolemic FA (h), mainly MUFA and PUFA, which tends to lower cholesterol, to hypercholesterolemic FA (H), primarily SFA, which tends to raise cholesterol [46]. In the present study, AI and TI were higher, while hH ratio was lower in the 0.71% diet compared to the remaining diets. This was mainly associated with the previously discussed higher content of SFA in the muscle of these fish, namely myristic acid (C14:0) and palmitic acid (C16:0).

Previous studies reported that higher rearing temperature tends to decrease muscle PUFA content and increase SFA and MUFA content [83,84,85]. This is usually due to the lower need for n-3 PUFA to maintain cell membrane fluidity and its use as a metabolic energy source to meet the physiological demands of elevated water temperatures [86]. However, the temperature impact on muscle FA composition depends strongly on diet and heat exposure duration [83]. In the present study, the 7-day heat challenge did not significantly alter the muscle FA composition of European seabass compared with the previous 180-day feeding period, suggesting that a 7-day thermal challenge similar to a heatwave did not significantly alter muscle lipid composition. Future studies should evaluate prolonged or repeated thermal challenges and investigate the underlying molecular mechanisms, including antioxidant, inflammatory, and lipid metabolism pathways, to better understand how dietary EPA+DHA may contribute to thermal-stress resilience in European seabass.

## 5. Conclusions

In conclusion, the present study indicates that a dietary EPA+DHA content of 0.56% (and a DHA/EPA ratio of 1.35/1) is adequate to meet the n-3 LC-PUFA requirements of European seabass juveniles (100–400 g) without compromising growth performance or health status, while allowing the recommended human daily intake of 250 mg of EPA+DHA to be met by consuming 100 g of fillet per day. Additionally, this dietary EPA+DHA level also seems sufficient to meet increased physiological needs during an acute temperature challenge.

## Figures and Tables

**Table 1:** Experimental diets composition (% dry weight).

Ingredients % Dry Weight Basis	EPA+DHA					
	0.56	0.66	0.71	0.9	1.11	1.27
Rapeseed oil ^1^	13.6	13.5	13.4	12.9	12.4	11.9
Salmon oil ^2^	2.5	2.5	2.5	2.5	2.5	2.5
Veramaris oil ^3^	0	0.125	0.25	0.75	1.25	1.75
Fish meal ^4^	10	10	10	10	10	10
Shrimp hydrolysate ^5^	2	2	2	2	2	2
Soy protein concentrate ^6^	25	25	25	25	25	25
Wheat gluten ^7^	15	15	15	15	15	15
Corn gluten meal ^8^	7	7	7	7	7	7
Sunflower meal ^9^	5	5	5	5	5	5
Whole wheat ^10^	10.9	10.9	10.9	10.9	10.9	10.9
Pea starch ^11^	3	3	3	3	3	3
Monoammonium phosphate ^12^	1.6	1.6	1.6	1.6	1.6	1.6
Calcium biphosphate ^13^	0.9	0.9	0.9	0.9	0.9	0.9
Lysine ^14^	1.2	1.2	1.2	1.2	1.2	1.2
Methionine ^15^	0.4	0.4	0.4	0.4	0.4	0.4
Vitamin premix ^16^	1	1	1	1	1	1
Mineral premix ^17^	0.6	0.6	0.6	0.6	0.6	0.6
Betaine ^18^	0.3	0.3	0.3	0.3	0.3	0.3
Proximate analysis (% DM)						
Dry matter, DM	95.8	96.9	98.3	97.0	95.9	97.3
Protein	47.4	47.1	47.3	46.9	47.3	47.7
Lipid	16.5	17.9	17.4	17.2	18.4	18.3
Ash	7.5	7.4	7.5	7.6	7.6	7.1
Gross energy (kJ g^−1^)	22.6	21.1	21.3	20.5	22.1	22.3

^1^ Sorgal, S.A. Ovar, Portugal. ^2^ Sorgal, S.A. Ovar, Portugal. ^3^ Veramaris (EPA: 16.71% FA; DHA: 48.8% FA); Delft, Netherlands. ^4^ Fish meal (74.9% CP, 10.7% CL); Sorgal, S.A. Ovar, Portugal. ^5^ Shrimp hydrolysate (*L. vannamei* trimmings: 73% CP, 12.1% CL); Sorgal, S.A. Ovar, Portugal. ^6^ Soy protein concentrate (55.5% CP, 0.97% CL); Sorgal, S.A. Ovar, Portugal. ^7^ Wheat gluten (78.3% CP, 2.18% CL); Sorgal, S.A. Ovar, Portugal. ^8^ Corn gluten meal (67.4% CP, 1.14% CL); Sorgal, S.A. Ovar, Portugal. ^9^ Sunflower meal (35.8% CP, 1.26% CL); Sorgal, S.A. Ovar, Portugal. ^10^ Whole wheat (10.63% CP, 2.16% CL); Sorgal, S.A. Ovar, Portugal. ^11^ Pea starch (93.5% DM); Sorgal, S.A. Ovar, Portugal. ^12^ Sorgal, S.A. Ovar, Portugal. ^13^ Sorgal, S.A. Ovar, Portugal. ^14^ Sorgal, S.A. Ovar, Portugal. ^15^ Sorgal, S.A. Ovar, Portugal. ^16^ Vitamin premix (mg kg^−1^ diet): retinol, 18,000 (IU kg^−1^ diet); calciferol, 2000 (IU kg^−1^ diet); alpha tocopherol, 35; menadion sodium bis., 10; thiamin, 15; riboflavin, 25; Ca pantothenate, 50; nicotinic acid, 200; pyridoxine, 5; folic acid, 10; cyanocobalamin, 0.02; biotin, 1.5; ascorbyl monophosphate, 50; inositol, 400. Premix, Viana do Castelo, Portugal. ^17^ Mineral premix (mg kg^−1^ diet): cobalt sulphate, 1.91; copper sulphate, 19.6; iron sulphate, 200; sodium fluoride, 2.21; potassium iodide, 0.78; magnesium oxide, 830; manganese oxide, 26; sodium selenite, 0.66; zinc oxide, 37.5; dicalcium phosphate, 8.02 (g kg^−1^ diet); potassium chloride, 1.15 (g kg^−1^ diet); sodium chloride, 0.4 (g kg^−1^ diet). ^18^ Sorgal, S.A. Ovar, Portugal.

**Table 2:** Fatty acid composition of the experimental diets.

	EPA+DHA					
Fatty acids (mg g^−1^)	0.56	0.66	0.71	0.9	1.11	1.27
SFA						
14:0	1.2	1.3	1.3	1.4	1.4	1.3
15:0	0.2	0.2	0.2	0.2	0.2	0.2
16:0	11.6	12.5	12.6	13.1	13.7	13.4
17:0	0.2	0.2	0.2	0.2	0.2	0.2
18:0	3.1	3.4	3.3	3.3	3.2	3.1
20:0	0.5	0.5	0.5	0.5	0.5	0.5
22:0	0.3	0.3	0.3	0.3	0.3	0.3
MUFA						
16:1 (n9+n7)	2.0	2.1	2.1	2.1	2.1	1.9
18:1 n9	56.5	59.4	59.8	57.6	56.4	51.9
18:1 n7	3.4	3.5	3.5	3.4	3.3	3.0
18:1 n5	0.1	0.1	0.1	0.1	0.1	0.1
20:1 (n11+n9+n7)	2.0	2.2	2.2	2.1	2.1	1.9
PUFA						
18:2 n6 (LA)	28.7	29.8	30.1	29	29.0	27.0
18:3 n3 (ALA)	8.5	8.9	8.9	8.7	8.5	7.8
20:4 n6 (ARA)	0.3	0.3	0.3	0.4	0.5	0.6
20:5 n3 (EPA)	2.4	2.7	2.8	3.3	3.9	4.2
22:5 n3 (DPA)	0.4	0.5	0.5	0.5	0.7	0.7
22:6 n3 (DHA)	3.2	4.0	4.3	5.7	7.3	8.5
∑SFA	17.1	18.5	18.6	19.0	19.6	19.1
∑MUFA	65.1	68.4	68.8	66.4	65.2	59.9
∑PUFA	45.4	48.2	49.0	50.0	52.3	51.4
∑n-3	15.8	17.3	17.8	19.6	21.8	22.7
∑n-6	29.4	30.7	31.0	30.2	30.4	28.5
*n-3/n-6 ratio*	0.5	0.6	0.6	0.7	0.7	0.8
DHA/EPA	1.3	1.5	1.5	1.7	1.9	2.0
EPA+DHA	5.6	6.6	7.1	9.0	11.1	12.7

SFA: saturated fatty acid; MUFA: monounsaturated fatty acid; PUFA: polyunsaturated fatty acid; LA: linoleic acid; ALA: α-linolenic acid; ARA: arachidonic acid; EPA: eicosapentaenoic acid; DPA: docosapentaenoic acid; DHA: docosahexaenoic acid.

**Table 3:** Growth performance and feed utilization efficiency of European seabass fed the experimental diets for 180 days at 23 °C.

	EPA+DHA							Polynomial Contrasts		
	0.56	0.66	0.71	0.9	1.11	1.27	SEM	ANOVA	Linear	Quadratic
Growth										
Zootechnical performance										
IBW (g)	107.0	107.0	107.0	107.0	107.0	107.0	0.01	0.366	0.424	0.115
FBW (g)	464.7	450.6	465.8	458.8	457.8	467.4	16.46	0.976	0.844	0.728
WG (g kg ABW^−1^ day^−1^) ^1^	7.0	6.8	7.0	6.9	6.9	7.0	0.12	0.973	0.859	0.688
FI (g kg ABW^−1^ day^−1^) ^2^	10.0	10.3	10.1	10.3	10.0	9.8	0.12	0.052	0.073	0.016
FE ^3^	0.69	0.66	0.68	0.67	0.69	0.71	0.01	0.269	0.224	0.068
DGI ^4^	1.66	1.62	1.67	1.65	1.64	1.67	0.05	0.976	0.818	0.727
TGC ^5^	0.07	0.07	0.07	0.07	0.07	0.07	0.00	0.840	0.264	0.603
PER ^6^	1.46	1.41	1.45	1.43	1.46	1.49	0.03	0.496	0.291	0.191
Survival Rate (%)	100	97.2	100	100	100	97.2	1.60	0.571	0.686	0.464
Organosomatic indices										
HSI (%) ^7^	1.5	1.6	1.7	1.6	1.4	1.4	0.1	0.077	0.115	0.080
VSI (%) ^8^	9.6	11.1	10.3	11.0	9.7	10.2	0.4	0.088	0.880	0.079

Data are presented as means (*n* = 3). SEM: pooled standard error of the mean. Average body weight (ABW): (initial body weight (IBW) + final body weight (FBW))/2. ^1^ Weight gain (WG): WG (g)/ABW (g)/trial duration (days) × 1000, where weight gain (WG, g fish^−1^) = IBW (g) − FBW (g). ^2^ Daily growth index (DGI): [(final weight^1/3^ − initial weight^1/3^)/time in days] × 100. ^3^ Feed intake (FI): dry feed intake/average body weight/days. ^4^ Feed efficiency (FE): wet weight gain/dry feed intake. ^5^ Thermal growth coefficient (TGC): (FBW^1/3^ − IBW^1/3^)/(temperature × time in days)) × 100. ^6^ Protein efficiency ratio (PER): weight gain/protein intake. ^7^ Hepatosomatic index (HSI): (liver weight/body weight) × 100. ^8^ Visceral somatic index (VSI): (viscera weight/body weight) × 100.

**Table 4:** Hematological and plasma parameters of European seabass fed the experimental diets for 180 days at 23 °C and subjected to an acute temperature challenge for 7 days.

	EPA+DHA						
	0.56	0.66	0.71	0.9	1.11	1.27	SEM
Growth trial							
Hemoglobin (g dL ^−1^)	3.2	3.0	2.8	3.5	3.2	3.1	0.2
Hematocrit (%)	38.2	33.1	33.8	31.5	28.5	34.3	2.0
MCHC (g 100 mL^−1^)	8.4	9.0	8.5	13.2	11.6	9.0	0.4
Glucose (mg dL ^−1^)	103.0	90.0	92.5	99.3	90.9	90.5	2.1
Lactate (mg dL ^−1^)	30.3	34.6	30.3	31.9	28.8	38.3	2.8
Peroxidase (U mL ^−1^)	30.5	28.4	32.6	30.3	34.0	38.2	1.3
Lysozyme (µg mg ^−1^ protein)	631.6	667.2	674.1	665.2	613.8	694.3	17.0
ACH50 (U mL ^−1^)	34.8	42.1	42.0	32.9	42.4	34.9	1.4
Thermal challenge							
Hemoglobin (g dL ^−1^)	3.4	3.8	3.7	3.8	3.6	3.8	0.3
Hematocrit (%)	31.6	34.4	32.0	28.1	31.5	30.7	1.7
MCHC (g 100 mL^−1^)	10.7	11.4	11.6	14.2	11.6	12.7	0.5
Glucose (mg dL ^−1^)	115.8	107.8	119.7	105.2	110.0	115.2	2.4
Lactate (mg dL ^−1^)	39.0	36.4	41.3	33.5	39.8	38.8	1.8
Peroxidase (U mL ^−1^)	60.6	60.2	69.6	53.5	62.7	62.7	1.4
Lysozyme (µg mg ^−1^ protein)	596.5	611.4	498.8	646.0	699.3	637.0	22.6
ACH50 (U mL ^−1^)	41.9	40.1	44.5	45.7	49.6	42.2	1.8
Two-way ANOVA	Variance source					Polynomial contrasts	
	Diet	Temperature		Interaction		Linear	Quadratic
Hemoglobin	0.390	<0.001		0.392		0.622	0.674
Hematocrit	0.008	0.014		0.250		0.008	0.009
MCHC	0.002	0.001		0.410		0.017	0.028
Glucose	0.402	<0.001		0.391		0.320	0.417
Lactate	0.933	0.070		0.833		0.683	0.531
Peroxidase	0.078	<0.001		0.271		0.138	0.449
Lysozyme	0.562	0.130		0.203		0.191	0.669
ACH50	0.241	0.007		0.432		0.596	0.169
Diets	0.56	0.66	0.71	0.9	1.11	1.27	
Hematocrit	b	ab	ab	a	a	ab	
MCHC	a	a	a	b	ab	a	

Data are presented as mean (*n* = 9). SEM: pooled standard error of the mean. Means within the same line, separated by different lowercase letters, indicate significant differences (*p* < 0.05) in EPA+DHA levels. MCHC: mean corpuscular hemoglobin concentration.

**Table 5:** Hepatic antioxidant enzyme activities and lipid peroxidation of European seabass fed the experimental diets at 23 °C and subjected to an acute temperature challenge for 7 days.

	EPA+DHA						
	0.56	0.66	0.71	0.9	1.11	1.27	SEM
Growth trial							
CAT (U mg protein^−1^)	411.6	282.3	277.3	334.3	283.0	213.6	19.8
SOD (U mg protein^−1^)	91.7	54.4	55.8	88.4	73.6	53.3	5.7
GR (mU mg protein^−1^)	1.3	1.4	1.1	1.8	1.9	1.1	0.1
GPX (mU mg protein^−1^)	2.6 ^A^	5.6 ^A^	3.9	4.7 ^A^	6.1	2.9	0.5
LPO (MDA g^−1^ tissue)	8.4	7.0	6.4	9.2	10.8	9.1	0.6
Thermal challenge							
CAT (U mg protein^−1^)	322.8	382.0	340.2	284.9	335.5	310.7	17.3
SOD (U mg protein^−1^)	95.3	80.3	66.0	94.3	74.7	98.1	5.7
GR (mU mg protein^−1^)	2.5	2.7	2.2	3.5	2.0	1.9	0.2
GPX (mU mg protein^−1^)	11.8 ^bB^	12.6 ^bB^	6.6 ^a^	11.8 ^bB^	7.7 ^ab^	6.3 ^a^	0.6
LPO (MDA g^−1^ tissue)	14.2	7.4	12.9	10.3	7.0	13.3	0.9
Two-way ANOVA	Variance source				Polynomial contrasts		
	Diet	Temperature		Interaction	Linear		Quadratic
CAT	0.343	0.266		0.186	0.030		0.913
SOD	0.137	0.059		0.569	0.638		0.288
GR	0.005	<0.001		0.193	0.479		0.022
GPX	0.001	<0.001		0.006	0.015		0.120
LPO	0.233	0.026		0.057	0.667		0.116
Diets	0.56	0.66	0.71	0.9	1.11	1.27	
GR	ab	ab	a	b	ab	a	

CAT: Catalase, SOD: Superoxide dismutase; GR: Glutathione reductase; GPX: Glutathione peroxidase: LPO: lipid peroxidation. Data are presented as mean (*n* = 9). SEM: pooled standard error of the mean. Means within the same line with different superscript lowercase letters indicate significant differences (*p* < 0.05) between EPA+DHA level and superscript uppercase letters for temperature, respectively. When a significant interaction was observed, temperature effects were analyzed using one-way ANOVA across increasing EPA+DHA levels.

**Table 6:** Muscle fatty acid composition and lipid quality indices (% of total fatty acids) of European seabass fed the experimental diets for 180 days at 23 °C.

	Growth Trial								Thermal Challenge							
	EPA+DHA								EPA+DHA							
	0.56	0.66	0.71	0.9	1.11	1.27	SEM	ANOVA	0.56	0.66	0.71	0.9	1.11	1.27	SEM	ANOVA
SFA																
14:0	1.1 ^a^	1.1 ^a^	1.5 ^b^	1.0 ^a^	1.1 ^a^	1.1 ^a^	0.04	0.001	1.1 ^a^	1.2 ^a^	1.5 ^b^	1.1 ^a^	1.1 ^a^	1.1 ^a^	0.03	0.001
15:0	0.18 ^a^	0.17 ^a^	0.23 ^b^	0.17 ^a^	0.18 ^a^	0.19 ^a^	0.01	0.001	0.18 ^a^	0.17 ^a^	0.23 ^b^	0.19 ^a^	0.18 ^a^	0.19 ^a^	0.00	0.001
16:0	12.2 ^a^	12.3 ^a^	14.0 ^b^	12.3 ^a^	12.3 ^a^	13.2 ^ab^	0.2	0.001	12.3	12.6	13.4	12.3	13.2	12.7	0.3	0.159
17:0	0.18 ^a^	0.18 ^a^	0.24 ^b^	0.18 ^a^	0.18 ^a^	0.19 ^a^	0.00	0.001	0.18 ^a^	0.19 ^a^	0.24 ^b^	0.19 ^a^	0.19 ^a^	0.19 ^a^	0.00	0.001
18:0	3.1 ^ab^	3.2 ^ab^	3.3 ^b^	3.2 ^ab^	2.8 ^a^	3.1 ^ab^	0.1	0.038	2.9 ^ab^	3.2 ^b^	3.2 ^b^	2.8 ^a^	3.0 ^ab^	3.0 ^ab^	0.06	0.015
20:0	0.2	0.3	0.2	0.3	0.3	0.2	0.03	0.483	0.3	0.3	0.3	0.3	0.3	0.3	0.00	0.473
22:0	0.11	0.12	0.10	0.11	0.10	0.09	0.01	0.142	0.1	0.1	0.1	0.1	0.1	0.1	0.00	0.831
MUFA																
16:1	2.3 ^a^	2.4 ^a^	2.9 ^b^	2.2 ^a^	2.3 ^a^	2.3 ^a^	0.06	0.001	2.3 ^a^	2.3 ^a^	3.0 ^b^	2.3 ^a^	2.2 ^a^	2.3 ^a^	0.06	0.001
18:1 n-9	42.5 ^cd^	43.3 ^d^	38.5 ^a^	41.0 ^bc^	40.6 ^b^	38.8 ^a^	0.4	0.001	42.4 ^b^	42.6 ^b^	39.4 ^a^	41.9 ^ab^	41.6 ^ab^	39.4 ^a^	0.6	0.004
18:1 n-7	2.5	2.5	2.5	2.4	2.4	2.3	0.03	0.052	2.4 ^ab^	2.4 ^ab^	2.5 ^b^	2.4 ^ab^	2.3 ^a^	2.3 ^a^	0.03	0.005
20:1	2.3 ^bc^	2.2 ^abc^	2.4 ^c^	2.1 ^ab^	2.1 ^ab^	2.1 ^a^	0.05	0.002	2.2 ^a^	2.2 ^a^	2.5 ^b^	2.2 ^a^	2.2 ^a^	2.2 ^a^	0.05	0.003
PUFA																
18:2 n-6 (LA)	17.0 ^c^	16.5 ^c^	15.2 ^a^	16.2 ^abc^	16.5 ^bc^	15.4 ^ab^	0.2	0.001	17.0	16.8	15.3	16.6	15.3	15.6	0.4	0.016
18:3 n-3 (ALA)	4.6 ^c^	4.5 ^c^	3.9 ^a^	4.4 ^bc^	4.5 ^c^	4.2 ^b^	0.04	0.001	4.6 ^b^	4.6 ^b^	4.0 ^a^	4.5 ^ab^	4.1 ^ab^	4.2 ^ab^	0.1	0.005
20:4 n-6 (ARA)	0.3 ^ab^	0.3 ^a^	0.4 ^bc^	0.5 ^c^	0.4 ^c^	0.6 ^d^	0.02	0.001	0.3 ^ab^	0.3 ^a^	0.4 ^ab^	0.4 ^abc^	0.5 ^bc^	0.5 ^c^	0.03	0.002
20:5 n-3 (EPA)	1.8 ^a^	1.7 ^a^	2.4 ^b^	2.2 ^b^	2.3 ^b^	2.7 ^c^	0.05	0.001	1.8 ^a^	1.7 ^a^	2.3 ^bc^	2.0 ^ab^	2.2 ^b^	2.6 ^c^	0.08	0.001
22:5 n-3 (DPA)	0.47 ^a^	0.45 ^a^	0.65 ^b^	0.57 ^ab^	0.59 ^b^	0.67 ^b^	0.05	0.001	0.47 ^a^	0.47 ^a^	0.61 ^bc^	0.53 ^ab^	0.58 ^abc^	0.368 ^c^	0.004	0.001
22:6 n-3 (DHA)	4.4 ^a^	4.0 ^a^	5.6 ^b^	6.2 ^b^	6.3 ^b^	7.9 ^c^	0.2	0.001	4.4 ^a^	4.0 ^a^	5.1 ^ab^	5.4 ^ab^	6.4 ^bc^	7.6 ^c^	0.3	0.001
EPA+DHA	6.1 ^a^	5.7 ^a^	8.0 ^b^	8.4 ^b^	8.6 ^b^	10.6 ^c^	0.3	0.001	6.2 ^a^	5.7 ^a^	7.4 ^ab^	7.4 ^ab^	8.6 ^bc^	10.2 ^c^	0.3	0.001
∑SFA	18.2 ^a^	18.6 ^a^	20.8 ^b^	18.3 ^a^	17.9 ^a^	19.1 ^a^	0.3	0.001	18.3 ^a^	18.7 ^ab^	20.2 ^b^	18.1 ^a^	19.0 ^ab^	18.5 ^ab^	0.4	0.028
∑MUFA	48.6 ^d^	49.2 ^d^	45.0 ^ab^	46.8 ^c^	46.4 ^bc^	44.4 ^a^	0.4	0.001	48.4 ^b^	48.6 ^b^	46.0 ^ab^	47.7 ^ab^	47.3 ^ab^	45.1 ^a^	0.6	0.010
∑PUFA	29.8 ^ab^	28.8 ^a^	30.0 ^ab^	31.6 ^bc^	32.2 ^c^	33.1 ^c^	0.5	0.001	29.9 ^ab^	29.3 ^a^	29.5 ^ab^	31.1 ^ab^	30.5 ^ab^	33.0 ^b^	0.7	0.043
∑n-3	12.0 ^a^	11.4 ^a^	13.7 ^b^	14.2 ^b^	14.6 ^b^	16.3 ^c^	0.3	0.001	12.1 ^ab^	11.6 ^a^	13.1 ^ab^	13.2 ^ab^	14.1 ^bc^	16.0 ^c^	0.5	0.001
∑n-6	17.6 ^b^	17.1 ^b^	15.9 ^a^	17.1 ^b^	17.4 ^b^	16.6 ^ab^	0.2	0.003	17.6 ^b^	17.6 ^b^	16.1 ^a^	17.6 ^b^	16.2 ^ab^	16.7 ^ab^	0.4	0.048
n-3/n-6 ratio	0.7 ^a^	0.7 ^a^	0.9 ^b^	0.8 ^b^	0.8 ^b^	1.0 ^c^	0.02	<0.001	0.7 ^a^	0.7 ^a^	0.8 ^b^	0.8 ^b^	0.9 ^b^	1.0 ^c^	0.04	<0.001
AI ^1^	0.2 ^a^	0.2 ^a^	0.3 ^b^	0.2 ^a^	0.2 ^a^	0.2 ^a^	0.01	<0.001	0.21 ^a^	0.22 ^a^	0.26 ^b^	0.21 ^a^	0.23 ^a^	0.22 ^a^	0.01	0.002
TI ^2^	0.06 ^a^	0.06 ^ab^	0.07 ^b^	0.06 ^a^	0.06 ^a^	0.06 ^ab^	0.01	0.012	0.06 ^ab^	0.06 ^ab^	0.07 ^b^	0.06 ^a^	0.06 ^ab^	0.06 ^ab^	0.01	0.061
hH ^3^	5.4 ^b^	5.3 ^b^	4.3 ^a^	5.4 ^b^	5.3 ^b^	4.9 ^b^	0.22	<0.001	5.3 ^b^	5.1 ^ab^	4.5 ^a^	5.3 ^b^	5.0 ^ab^	5.1 ^ab^	0.18	0.015
PI ^4^	81.7 ^a^	77.2 ^a^	95.4 ^b^	99.8 ^b^	102.4 ^b^	117.1 ^c^	3.9	<0.001	82.2 ^a^	78.5 ^a^	90.8 ^ab^	92.4 ^ab^	99.9 ^bc^	114.4 ^c^	6.6	<0.001
NC_250_ (%DI) ^5^	139.5 ^a^	139.1 ^a^	172.4 ^ab^	142.1 ^a^	212.4 ^b^	230.1 ^b^	23.1	0.001	145.7 ^a^	137.6 ^a^	183.8 ^ab^	170.9 ^ab^	194.7 ^ab^	247.9 ^b^	17.7	0.005
Two-way ANOVA		Variance source						Polynomial contrasts			Diets					
		Diet		Temperature		Interaction		Linear	Quadratic		0.56	0.66	0.71	0.9	1.11	1.27
∑SFA		<0.001		0.843		0.143		0.999	0.008		a	a	b	a	a	a
∑MUFA		<0.001		0.134		0.443		<0.001	0.771		e	de	ab	cd	bc	a
∑PUFA		<0.001		0.319		0.597		<0.001	0.020		ab	a	ab	bc	bc	c
20:5 n-3 (EPA)		<0.001		0.067		0.582		<0.001	0.430		a	a	c	b	bc	e
22:6 n-3 (DHA)		<0.001		0.140		0.615		<0.001	0.006		a	a	b	bc	c	e
EPA+DHA		<0.001		0.133		0.619		<0.001	0.014		a	a	ab	ab	bc	c

SFA: saturated fatty acid; MUFA: monounsaturated fatty acid; PUFA: polyunsaturated fatty acid; LA: linoleic acid; ALA: α-linolenic acid; ARA: arachidonic acid; EPA: eicosapentaenoic acid; DPA: docosapentaenoic acid; DHA: docosahexaenoic acid. Values are presented as mean (*n* = 9) and SEM (pooled standard error of the mean). Means within the same line with different superscript lowercase letters indicate significant differences (p < 0.05) between EPA+DHA level. ^1^ AI: atherogenicity index: (C12:0 + (4 × C14:0) + C16:0)/(ΣMUFA + Σn-3 + Σn-6). ^2^ TI: thrombogenicity index: (C14:0 + C16:0 + C18:0)/((0.5 × ΣMUFA) + (0.5 × Σn-6) + (3 × Σn-3) + ratio n-3/n-6). ^3^ hH: hypo/hypercholesterolemic ratio: (C18:1n-9 + C18:2n-6 + C20:4n-6 + C18:3n-3 + C20:5n-3 + C22:5n-3 + C22:6n-3)/(C14:0 + C16:0). ^4^ PI: peroxidation index: 0.025 × (% Σmonoenoic FA) + 1 × (% Σdienoic FA) + 2 × (% Σtrienoic FA) + 4 × (% Σtetraenoic FA) + 6 × (% Σpentaenoic FA) + 8 × (% Σhexaenoic FA). ^5^ NC_250_: nutritional contribution of EPA+DHA, considering a meal portion of 100 g, as % of the daily intake (DI) of 250 mg day^−1^ for the maintenance of cardiovascular health in adults [43]; NC_250_ = (((EPA+DHA) × muscle DM)/250) × 1000.

## Data Availability

The data presented in the present study are available upon request from the corresponding author. During the preparation of this work, the authors used Grammarly to improve the language and grammar of the manuscript. After using this tool, the authors reviewed and edited the content as needed and took full responsibility for the content of the published article.
